# Diagnostic innovations in Equine Parasitology: a Nanogold-ELISA for sensitive serodiagnosis of migratory *strongylus vulgaris* larvae infections

**DOI:** 10.1186/s12917-024-04389-x

**Published:** 2024-12-27

**Authors:** Hanadi B. A. Baghdadi, Mohamed Abdelsalam, Marwa M. Attia

**Affiliations:** 1 Biology Department, College of Science, Imam Abdul Rahman Bin Faisal University, Dammam, 31441 Saudi Arabia; 2 Department of Aquatic Animal Medicine and Management, Faculty of Veterinary Medicine, Cairo University, Giza, 12211 Egypt; 3 Department of Parasitology, Faculty of Veterinary Medicine, Cairo University, Giza, 12211 Egypt

**Keywords:** *Strongylus vulgaris*, Indirect-ELISA, Nano-based ELISA, Nanotechnology, Verminous aneurysms

## Abstract

*Strongylus vulgaris*, a devastating parasitic nematode in equids, causes life-threatening verminous aneurysms that are challenging to diagnose early. This study pioneered integrating nanotechnology into an indirect enzyme-linked immunosorbent assay (i-ELISA) system to enhance the sensitivity and specificity for detecting *S. vulgaris* larval antigens in equine serum samples, with PCR confirmation of the species. A conventional i-ELISA and an innovative nano-based ELISA were developed using excretory-secretory antigens from adult *S. vulgaris* worms. The nano-ELISA incorporated gold nanoparticles (17.4–41.4 nm) conjugated with detection antibodies, enabling remarkable signal amplification. Of the 120 examined equines, 100 (83.33%) were positive for *S. vulgaris* infection. A conventional i-ELISA and an innovative nano-ELISA incorporating 17.4–41.4 nm gold nanoparticles were optimized using *S. vulgaris* excretory-secretory antigens. Both assays demonstrated high specificity, with no cross-reactivity against sera from animals infected with other helminth parasites. Remarkably, optical density (OD) readings from both i-ELISAs exhibited a positive quantitative correlation with infection intensity. The i-ELISA OD ranged from 0.45–0.74 (G3), 0.75–0.94 (G2), to 0.95–2.5 (G1). The nano-ELISA showed enhanced signal amplification, with OD ranging from 0.40–0.84 (G3), 0.85–0.99 (G2), to 1.0–3.5 (G1). This nanotechnology-amplified ELISA opens new, highly sensitive, and specific techniques for parasitic diagnosis in equine medicine. Its superior performance, facilitated by signal-amplifying gold nanoparticles, illuminates nanotechnology's potential in revolutionizing parasitological diagnostics for enhanced animal health and welfare management.

## Introduction

*Strongylus vulgaris* (*S. vulgaris*) stands out as the most pathogenic and devastating among the diverse array of helminth parasites afflicting equids. These migratory larval nematodes induce a severe condition known as verminous aneurysms by traversing the cranial mesenteric artery and its branches. Their migrations trigger fibrinous endarteritis, leading to aneurysm formation and thrombosis in these major arterial vessels [1]. The consequent thromboembolism obstructs blood supply, culminating in intestinal segment ischemia, infarction, and a potentially fatal colic syndrome characterized by severe abdominal pain [2].

Conventional diagnostic methods face considerable challenges in detecting prepatent *S. vulgaris* infections. The morphological examination cannot differentiate the strongyle-type eggs shed by over 50 different nematode species in equids [3]. Techniques like copro-cultures [4, 5] and real-time PCR [6] are limited to identifying adult female worms in the intestine. Still, they cannot detect the pathogenic migratory larval stages during their 6-month prepatent period. This diagnostic gap has grave implications, as early intervention is crucial to prevent the development of life-threatening verminous aneurysms.

A recent breakthrough was the validation of an enzyme-linked immunosorbent assay (ELISA) that can detect circulating antigens from migratory *S. vulgaris* larvae [7]. However, the performance of this test may still be suboptimal for reliable early diagnosis, necessitating further enhancements. This is where nanotechnology offers promising solutions. Nanotechnology has revolutionized diagnostic assays by harnessing the unique properties of nanomaterials. Nanoparticles and nanostructured surfaces can amplify detection signals, enhance antigen–antibody interactions, enable multiplexing, and improve overall sensitivity and specificity [4, 7]. For instance, gold nanoparticles conjugated with detection antibodies can catalyze the deposition of opaque metals, intensifying colorimetric signals [5]. Nanostructured surfaces presenting antigens in high density facilitate more efficient antibody capture [5]. Fluorescent nanoparticles enable simultaneous multiplex detection of different analytes [6]. The present study pioneers the integration of such cutting-edge nanotechnology strategies to develop an enhanced i-ELISA system for early, sensitive, and specific detection of *S. vulgaris* larval antigens in serum samples from infected horses. Successful optimization of this nanotechnology-based diagnostic could enable timely therapeutic interventions, preventing progression to the devastating consequences of verminous aneurysms.

*S. vulgaris* remains a widespread and concerning parasite in horses globally, as evidenced by recent molecular epidemiological studies [8–10]. The impact of this parasite extends beyond the well-known verminous aneurysms, with recent research highlighting its potential to cause a wider range of pathological effects. Notably, *S. vulgaris* has been linked to previously unreported clinical presentations, such as non-strangulating intestinal infarctions [10]. This newfound association underscores the need for further investigation into the parasite's full spectrum of potential impacts on equine health. Furthermore, a study suggests a potential link between *S. vulgaris* prevalence and deworming practices that rely on selective therapies [11]. This finding highlights the potential value of improved diagnostic tools in refining parasite control programs to maximize their effectiveness in managing *S. vulgaris* infections in horses. By addressing these knowledge gaps through continued investigations, we can strive towards enhanced equine health and well-being through the development of more effective parasite control strategies.

In the past, diagnosing *S. vulgaris* infections in horses presented a significant challenge. Existing diagnostic methods had limitations, often hindering the early detection of this parasite. This delay in diagnosis could lead to severe complications like verminous aneurysms. This pioneering study aimed to revolutionize this field by developing a highly sensitive and specific i-ELISA utilizing nanotechnology for early diagnosis. This advanced approach held the potential to significantly improve *S. vulgaris* management, ultimately safeguarding the health and well-being of equine populations worldwide.

## Materials and methods

### Samples collection

From January to April 2023, a total of 120 horses from the Giza Zoo abattoir in Giza, Egypt, underwent postmortem examinations. During this process, *Strongylus* spp. nematodes were collected from the large intestines of the horses, and serum samples were obtained at the time of slaughter. The recovered *Strongylus* spp. specimens and serum samples were preserved and maintained on ice until their transfer to the Parasitology Laboratory. The serum samples were collected in sterile Eppendorf tubes and stored at -20 °C until further analysis. To establish control groups, the serum samples were classified based on the presence of other parasitic infections. Specifically, 10 serum samples were collected from horses infected solely with *Gasterophilus intestinalis* (*G. intestinalis*) larvae, 10 samples from horses infected with *Parascaris equorum* (*P. equorum*) (adult or larval stages), and 10 samples from horses harboring *Oxyuris equi* (*O. equi*) adults. Additionally, fecal samples were collected from all animals to determine the egg per gram count (EPG) [12] for each examined positive animal, enabling the assessment of infection intensity levels using the McMaster technique [13, 14]. This sampling approach was approved by the ethical committee of the Faculty of Veterinary Medicine, Cairo University*.*

### DNA extraction and pcr amplification

Three adult worm specimens were individually dissected into small pieces and transferred to separate Eppendorf tubes. Genomic DNA was isolated from each sample using the NucleoSpin Tissue mini kit (Macherey–Nagel GmbH & Co., Germany) according to the manufacturer's protocol. Following extraction, the concentration and purity of the extracted DNA were determined using a Nanodrop ND-2000 spectrophotometer (Thermofisher). The extracted DNA was stored at -20 °C for subsequent PCR amplification.

PCR reactions, with a total volume of 25 μl, were prepared using 12.5 μl Emerald Amp Max PCR Master Mix (Takara, Japan), 1 μl each of forward and reverse primers at 20 pmol concentration, 4 μl of DNA template, and 6.5 μl of nuclease-free water. The internal transcribed spacer 2 (ITS2) region of the ribosomal DNA was amplified using the forward primer NC1 (5'-ACGTCTGGTTCAGGGTTGTT-3') and reverse primer NC2 (5'-TTAGTTTCTTTTCCTCCGCT-3') [15]. Thermal cycling conditions were optimized based on a previous study and consisted of an initial denaturation step at 95 °C for 6 min, followed by 35 cycles of denaturation (94 °C for 45 s), annealing (60 °C for 90 s), and extension (72 °C for 60 s) [16]. A final extension step at 72 °C for 5 min ensured complete amplicon formation. The amplified PCR products were subsequently purified using the QIAquick PCR Purification Kit (Qiagen, USA) to remove any residual reaction components.

### Sequencing and phylogenetic analysis

The purified ITS2 amplicons were submitted for bi-directional Sanger sequencing (Macrogen Inc., Seoul, South Korea) using the same forward (NC1) and reverse (NC2) primers employed in the PCR amplification. This bi-directional approach ensures complete and accurate sequence coverage. Sequencing reactions were performed with the Big Dye Terminator Cycle Sequencing Kit. Electrophoresis was conducted on an Applied Biosystems 3730XL DNA Analyzer (USA) to obtain raw sequence data. The raw sequence data obtained from both forward and reverse reads were edited and assembled into a consensus sequence using BioEdit software [17]. The final consensus sequences were submitted to GenBank (NCBI) to acquire unique accession numbers. Nucleotide BLAST analysis of the consensus sequences was performed against the NCBI GenBank database to confirm the identity of the isolated *S. vulgaris* samples. To elucidate the evolutionary relationships between the isolated *S. vulgaris* samples and closely related species, a phylogenetic tree was constructed using the ITS2 sequence alignment in MEGA 11 [18]. The maximum likelihood (ML) method was employed with 1000 bootstrap replicates to evaluate the robustness of the inferred phylogenetic relationships.

### Preparation of *S. vulgaris* antigen

Twenty adult *S. vulgaris* nematodes were incubated for 6 h at 37 °C in Petri dishes containing 10 ml of phosphate-buffered saline (PBS) supplemented with 10,000 U/ml of penicillin–streptomycin (ThermoFisher) and protease inhibitors (Sigma-Fast). The following day, the PBS solution was collected and centrifuged at 14,000 rpm for 30 min at 4 °C. The resulting supernatant was dialyzed and concentrated using polyethylene glycol (PEG-6000; Sigma, USA). The concentrated solution contained excretory-secretory (E/S) antigens from S. vulgaris, which were stored at -20 °C for further analysis [19].

### Preparation of hyperimmune positive sera

To generate anti-*S. vulgaris* hyperimmune sera, six male albino rats (*Rattus norvegicus*) weighing approximately 200 g were used. The rats were divided into two groups: a control group of three rats and an immunized group of three rats. The rats were housed in standard rat cages with straw bedding, provided with regularly balanced rat pellets, and given unrestricted access to water. The temperature and humidity were maintained at suitable levels for the rats. The immunized group of rats received three doses of the *S. vulgaris* E/S antigen, with one subcutaneous (S/C) dose and two intramuscular (I/M) doses. Each dose consisted of 100 µg of protein from the E/S antigen emulsified with 1 ml of mineral oil.

### Checkerboard titration

The protein concentration of the E/S antigen was determined using the Lowry assay [20]. A checkerboard titration was performed to determine the optimal dilutions of the antigen and sera for the i-ELISA. Antigen concentrations of 10, 15, 30, 60, and 120 µg/ml were tested against serum dilutions of 1:50, 1:100, 1:200, 1:400, and 1:800 [21].

### Preparation of Gold nanoparticles

Gold nanoparticles, purchased from Nanotech Egypt®, were characterized using transmission electron microscopy (TEM). The nanoparticles were sonicated in ethanol and deposited onto a copper-coated carbon grid. After allowing the ethanol to evaporate, the nanoparticles were imaged using a Joel-JEM Japan 2100 TEM operating at 80 kV [19, 22].

### Serological methods

#### Indirect Enzyme-Linked Immunosorbent Assay (I-ELISA)

Flat-bottom 96-well i-ELISA plates were coated with *S. vulgaris* E/S antigen (15 µg/ml) in coating buffer (pH 9.6) and incubated overnight at 4 °C. The plates were then blocked with 200 μl/well of bovine serum albumin (BSA) in PBS at 37 °C for 2 h to prevent non-specific binding. After blocking, the plates were washed three times with PBS containing 0.05% Tween-20 (PBS-T). Serum samples were diluted 1:200 in PBS, and 100 μl of each diluted serum sample was added to three separate wells for triplicate measurements. The plates were incubated for 2 h at 37 °C. Following incubation, the plates were washed three times with PBS-T. Next, 100 μl of horseradish peroxidase-conjugated protein A (IgG conjugate; Sigma, A-5420) diluted 1:2000 in PBS was added to each well, and the plates were incubated for 1 h at 37 °C. After incubation, the plates were washed three times with PBS-T. The substrate solution, consisting of o-phenylenediamine (OPD; 10 mg) in citrate buffer (pH 5.0) with 30% hydrogen peroxide (H_2_O_2_), was added at 100 μl per well. The reaction was allowed to proceed until sufficient color development was observed, at which point it was stopped by adding 100 μl of 3N H_2_SO_4_ per well. The optical density (OD) of each well was measured at a wavelength of 450 nm using an i-ELISA reader (Bio-Rad, USA) [19, 20]. The cut-off value (COV) was calculated according to Carpenter [23], where the COV was determined as the mean OD of the negative control multiplied by twice the standard deviation. Samples with ODs equal to or higher than the COV were considered positive for *S. vulgaris*, while samples with ODs below the COV were considered negative.

#### Nano Gold-based ELISA

An enhanced nano-based ELISA (nano-ELISA) was performed using a similar protocol as the conventional i-ELISA, with a modification in the conjugation step. Gold nanoparticles (15–30 nm) were used in the conjugation phase by mixing 50 μl of the gold nanoparticles with 50 μl of the horseradish peroxidase-conjugated protein A (IgG conjugate; Sigma, A-5420) at a 1:2000 dilution [24]. To confirm that the nanoparticles were conjugated with the conjugate, TEM was used for characterization after conjugation of the gold nanoparticles, which showed large particles of nanoparticles within the nanosize.

#### Estimation of ELISA parameters

The performance of the two ELISA methods (indirect and nano-based) was evaluated by estimating various parameters, including specificity, sensitivity, negative and positive predictive values, and disease prevalence. The average OD readings were calculated, and the OD values of the tested and control sera were compared.

## Results

### Prevalence of *S. vulgaris* infection and intensity categorization

Of the 120 horses examined during the study period, postmortem examinations revealed that 100 (83.33%) were positive for *S. vulgaris* infection. The positive horses were further categorized into three groups based on the intensity of infection, as determined by the eggs per gram (EPG) counts in their fecal samples. Group 1 (G1) consisted of severely infected animals with EPG counts ranging from 1,500 to 2,500. Group 2 (G2) included moderately infected animals with EPG counts between 500 and 1,500. Group 3 (G3) comprised animals with low infections, having EPG counts lower than 500 (Table 1 and Table 2).

**Table 1:** Optical Densities (OD) of horses infected with *S. vulgaris* E/S

Group of Animals	No. of infected horses	Mean EPG	Optical Densities by indirect-ELISA	Optical Densities by nano-ELISA
G1	20	> 1500-2500	0.95-2.5	1-2.85
G2	35	> 500-1500	0.75-0.94	0.85-0.99
G3	45	To 500	0.45- 0.74	0.40- 0.84

**Table 2:** Results from Indirect ELISA and nano-ELISA

Tests	Indirect ELISA	nano-ELISA
Values		
True Positive	96	98
False Negative	4	2
False positive	0	0
True Negative	20	20

### Molecular identification of *S. vulgaris*

The ITS2 region of the ribosomal DNA was successfully amplified from three individual worms using the primer pair NC1 and NC2, yielding an amplicon size of approximately 267 bp. The obtained sequences were submitted to the GenBank database, and the following accession numbers were assigned: PP528643, PP528644, and PP528645. Sequence analysis revealed that the AT content of the ITS2 sequences was 66.29%, while the GC content was 33.71%. The average base composition was 33.33% for adenine (A), 17.98% for cytosine (C), 15.73% for guanine (G), and 32.96% for thymine (T). The alignment analysis confirmed that these nematodes belong to the genus *Strongylus* and were identified as *S. vulgaris*.

The three ITS2 sequences obtained from *S. vulgaris* were compared to investigate intraspecific variations. The pairwise sequence alignment revealed high similarity among the sequences, ranging from 99.62% to 99.81%. The sequence differences were attributed to a few nucleotide substitutions at specific positions within the ITS2 region. Sequence PP528643 differed from PP528644 by a single nucleotide substitution (C to A) at position 174. Compared to PP528645, PP528643 exhibited a single nucleotide difference (A to T) at position 177. Between PP528644 and PP528645, a single nucleotide substitution (C to T) was observed at position 171. These intraspecific variations highlight the existence of distinct haplotypes within the *S. vulgaris* population studied, reflecting the genetic diversity present within this species. However, the high overall similarity among the sequences supports their identification as belonging to the same species, *S. vulgaris*.

The BLAST analysis of the accession numbers PP528643, PP528644, and PP528645 showed a sequence similarity ranging from 99.38% to 95.45% when compared to previously reported *S. vulgaris* sequences (OP672317, MF489225, KP693439, X77863, OK235477). In contrast, the sequence similarity to *S. edentatus* (X77807, OP672312, OP672311, and MT193648) ranged from 74.74% to 72.41%. Furthermore, the sequence similarity to *S. equinus* (X77808) and *S. asini* (X99345) was 75.88% and 76.24%, respectively.

### Phylogenetic analysis

**Fig. 1:**
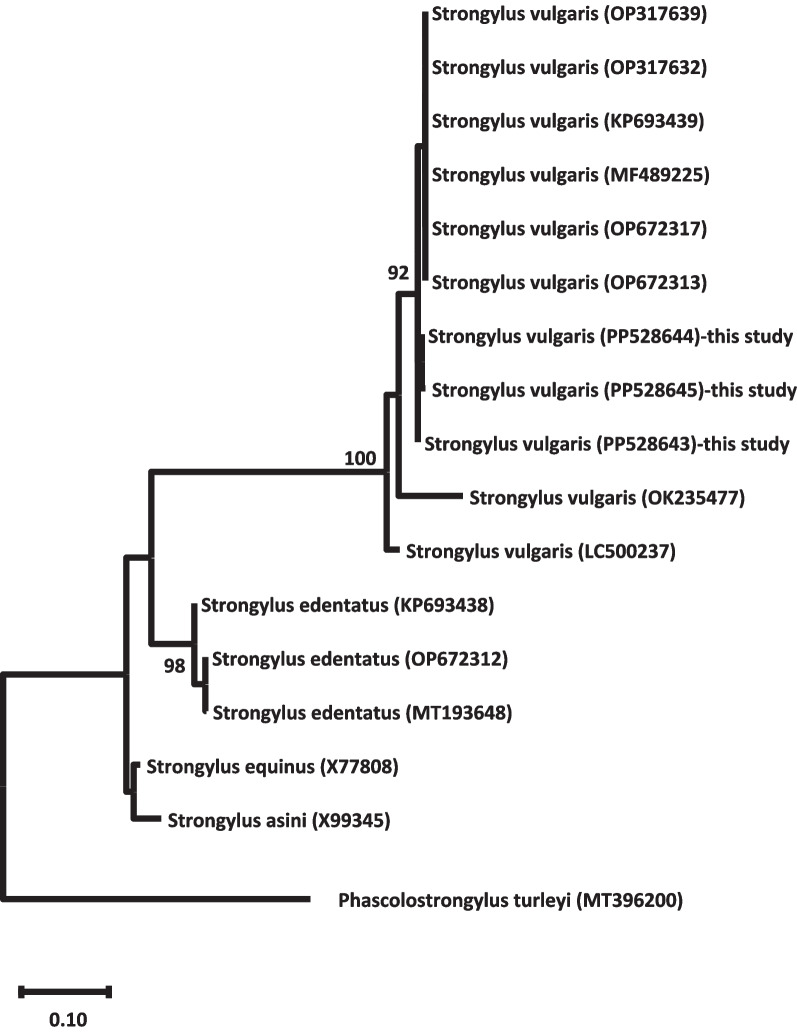
The phylogenetic tree based on the ITS2 region was constructed using the maximum likelihood method. The tree depicts the evolutionary relationship between *S. vulgaris* and related species. Bootstrap values are shown at the nodes, indicating the robustness of the branching patterns. The sequence from *Phascolostrongylus turleyi* (MT396200) was used as an outgroup to root the tree

The phylogenetic tree based on the maximum likelihood analysis of the ITS2 sequences revealed two major clades (Fig. 1). The first clade was subdivided into two subclades. All three sequences of *S. vulgaris* (PP528643, PP528644, and PP528645) obtained in this study formed a well-supported monophyletic group within the first subclade, with a bootstrap value of 100%. This subclade also included other previously reported *S. vulgaris* sequences, indicating the close evolutionary relationship among these isolates. The second subclade within the first major clade consisted of sequences belonging to the closely related species *S. edentatus*. The second major clade was further divided into two subclades, one containing sequences of *S. equinus* and the other comprising sequences of *S. asini*. The phylogenetic analysis clearly separated *S. vulgaris* from the other *Strongylus* spp., with strong bootstrap support for the respective clades and subclades. This topology suggested that *S. vulgaris* is a distinct and well-defined species within the genus *Strongylus*, exhibiting genetic divergence from its closely related counterparts; Fig. 1**.**

### Checkerboard titration and cut-off value determination

The checkerboard titration was performed to determine the optimal concentrations of the S. vulgaris excretory-secretory (E/S) antigen and serum dilutions for the indirect ELISA (i-ELISA) and the nano-based ELISA. For the i-ELISA, the optimal concentrations were determined to be 15 μg/ml of the E/S antigen and a serum dilution of 1:200. In contrast, the nano-based ELISA required lower concentrations, with optimal values of 10 μg/ml of the E/S antigen and a serum dilution of 1:400. The cut-off values were 0.45 and 0.40 for the i-ELISA and nano-based ELISA, respectively. Samples with optical density (OD) readings equal to or higher than the cut-off value were considered positive for *S. vulgaris* infection; this is the double of the mean of negative sera.

### Specificity and cross-reactivity

Both the i-ELISA and the nano-based ELISA demonstrated high specificity, as no cross-reactivity was observed when the E/S antigen was tested against sera from animals infected with other parasites, including *G. intestinalis*, *P. equorum*, or *O. equi*, in the absence of *S. vulgaris* infection. This result indicates that the E/S antigen specifically detects antibodies against *S. vulgaris* without interference from other parasitic infections. *ELISA Optical Density Readings and Infection Intensity.*

The OD readings obtained from the two ELISA methods were analyzed in relation to the intensity of S. vulgaris infection, as determined by the EPG counts.

### Indirect ELISA (i-ELISA)

In the i-ELISA, high OD values ranging from 0.95 to 2.5 were recorded for the severely infected G1 group, indicating a strong antibody response against the E/S antigen. The OD values for the moderately infected G2 group ranged from 0.75 to 0.94, while the lowest OD values, ranging from 0.45 to 0.74, were observed for the low-infection G3 group (Table 1). These results demonstrate a positive correlation between the OD readings and the intensity of *S. vulgaris* infection, as higher antibody levels were detected in animals with higher EPG counts.

### Nano-based ELISA

In the nano-based ELISA, the incorporation of gold nanoparticles resulted in enhanced signal amplification, leading to higher OD values compared to the i-ELISA. High OD values ranging from 1.0 to 3.5 were recorded for the severely infected G1 group. The OD values for the moderately infected G2 group ranged from 0.85 to 0.99, while the lowest OD values, ranging from 0.40 to 0.84, were observed for the low-infection G3 group (Table 1). Similar to the i-ELISA, a positive correlation between the OD readings and the intensity of *S. vulgaris* infection was observed in the nano-based ELISA.

### Characterization of Gold Nanoparticles before and after conjugation

**Fig.2:**
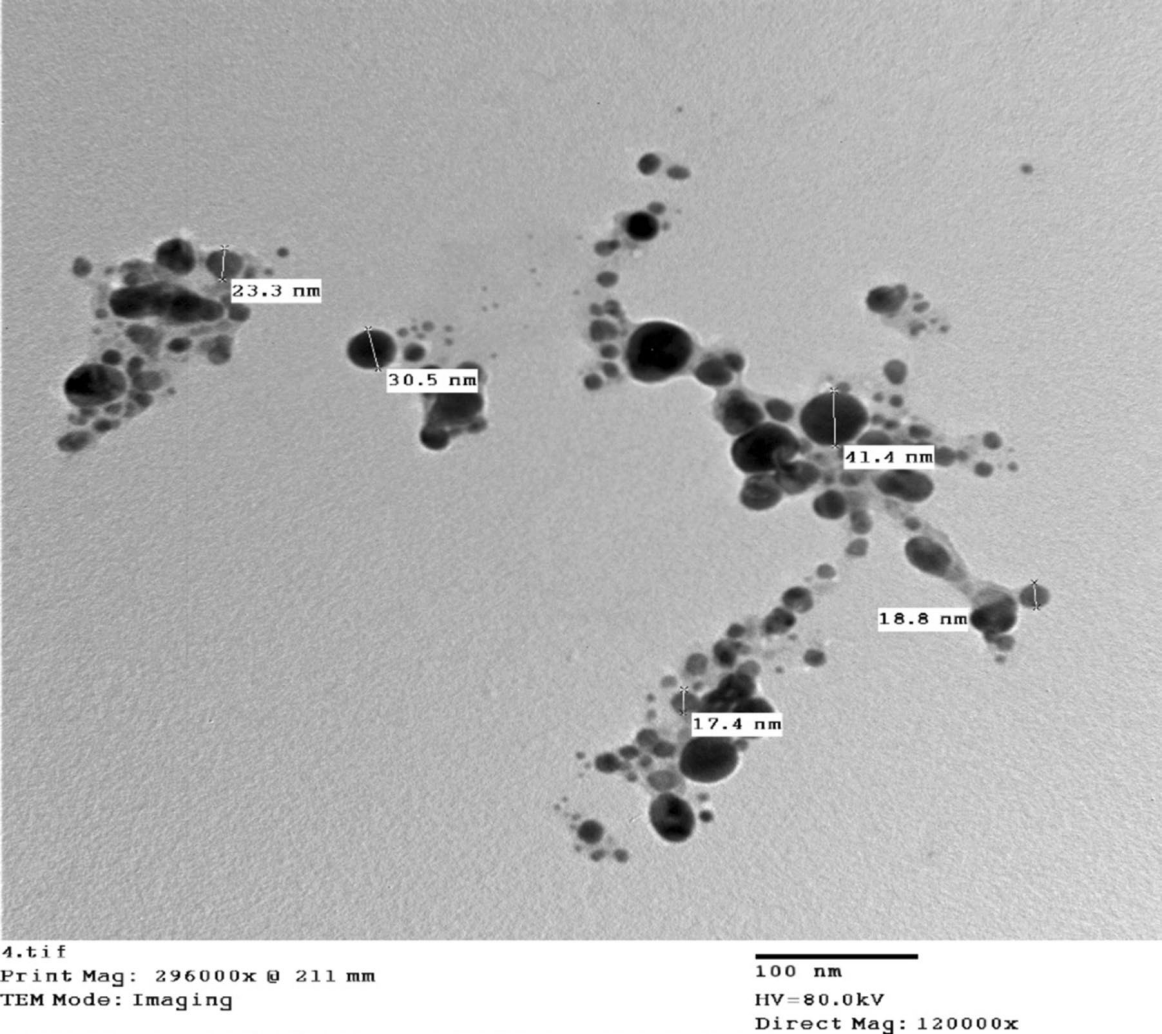
Transmission electron microscopy of gold nanoparticles reveals that their particle sizes range from 17.4 to 41.4 nm

**Fig.3:**
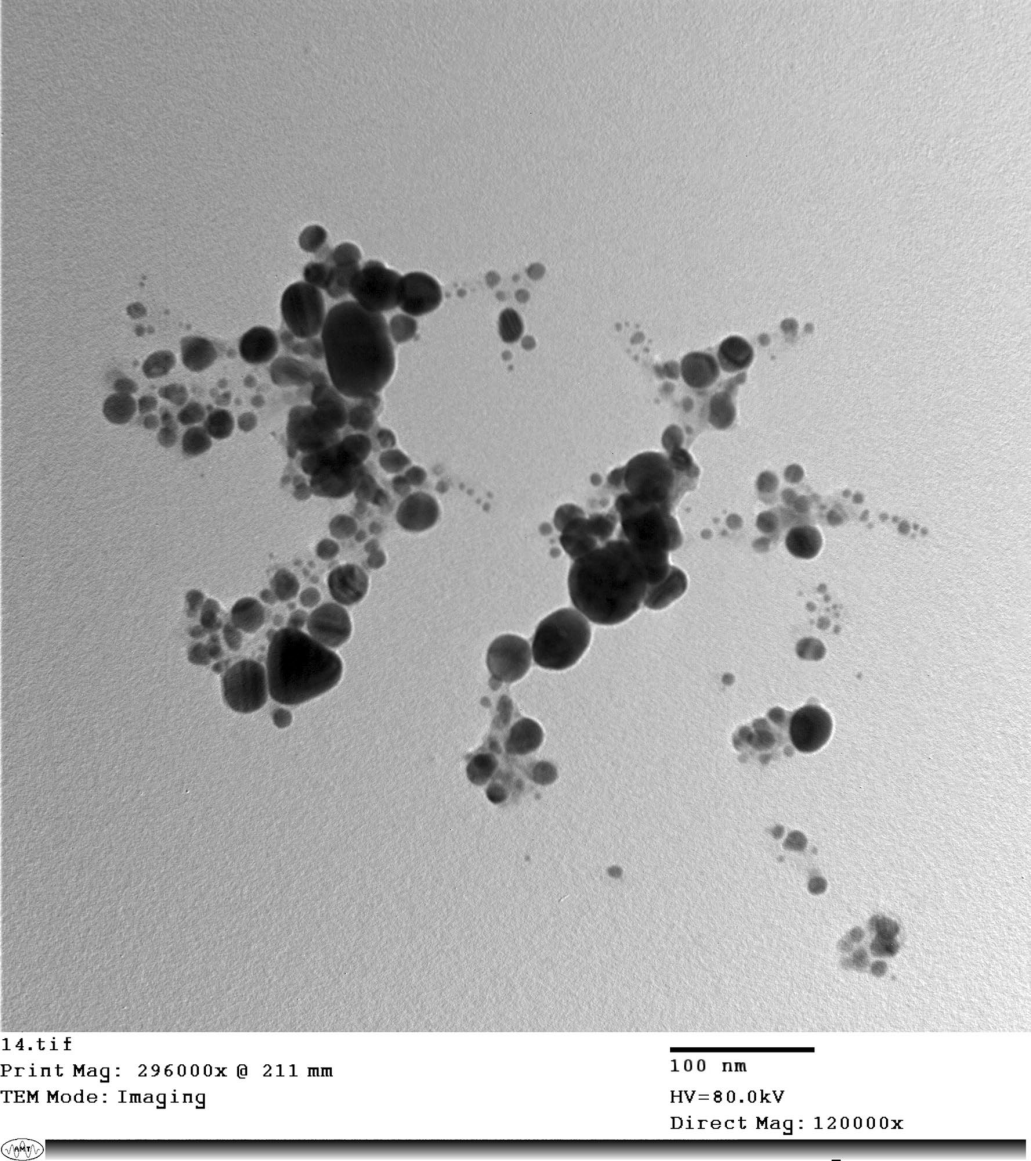
Transmission electron microscopy of gold nanoparticles after conjugation with the specific conjugate

Transmission electron microscopy (TEM) analysis was performed to characterize the gold nanoparticles used in the nano-based ELISA. The TEM micrographs revealed that the nanoparticles had a size range of 17.4 nm to 41.4 nm (Figs. 2 and 3). The incorporation of these nanoparticles in the ELISA system contributed to the enhanced signal amplification and improved performance compared to the conventional i-ELISA.

### Diagnostic Performance Evaluation

The diagnostic performance of the two ELISA methods was evaluated by determining their specificity, sensitivity, and accuracy (Table 3). The nano-based ELISA demonstrated higher specificity, sensitivity, and accuracy compared to the i-ELISA. These results indicate that the integration of nanotechnology into the ELISA system improved the overall diagnostic performance for the detection of *S. vulgaris* infection in horses.

**Table 3:** The diagnostic sensitivity and specificity of the two tests

Tests	Indirect-ELISA	Nano-ELISA
Statistics	Statistics (95% CI)	statistics 95% CI
Sensitivity	96.00% (90.07% to 98.90%)	98.00% (92.96% to 99.76%)
Specificity	100.00% (83.16%—100.00%)	100.00% (83.16% to 100.00%)
Positive likelihood Ratio	-	-
Negative likelihood Ratio	0.04 (0.02 to 0.10)	0.02 (0.01 to 0.08)
Disease Prevalence	83.33%	83.33%
Positive Predictive Value	100.00% (96.23% to 100.00%)	100.00% (96.31% to 100.00%)
Negative Predictive Value	83.34% (65.69% to 92.89%)	90.91% (71.72% to 97.53%)
Accuracy	96.67% (91.69% to 99.08%)	98.33% (94.11% to 99.80%)

## Discussion

The accurate diagnosis of *S. vulgaris* infection, particularly during the prepatent phase when immature larvae migrate through the cranial mesenteric artery, is crucial for timely therapeutic intervention and prevention of life-threatening verminous aneurysms. In this study, the molecular analysis of the ITS2 region successfully identified the nematodes as *S. vulgaris*, revealing distinct intraspecific variations among the obtained sequences. The intraspecies similarity analysis highlighted the presence of distinct haplotypes within the *S. vulgaris* population studied. Furthermore, the phylogenetic analysis based on the ITS2 sequences clearly separated *S. vulgaris* from other closely related species, forming a well-supported monophyletic clade. These findings provide valuable insights into the genetic diversity and evolutionary relationships within the genus *Strongylus*, further supporting the identification and delineation of *S. vulgaris* as a distinct species.

Conventional diagnostic methods, such as fecal egg counts and larval cultures, are limited in their ability to detect these migratory larval stages [25, 26]. The development of a sensitive and specific serological assay is therefore imperative for early diagnosis and effective management of this devastating parasitic disease. In the present study, we explored the application of nanotechnology to enhance the performance of an i-ELISA for the detection of *S. vulgaris* antigens in serum samples from infected horses. The integration of gold nanoparticles into the i-ELISA system resulted in improved sensitivity, specificity, and overall diagnostic accuracy compared to the conventional i-ELISA.

Nanotechnology has revolutionized the field of biomedical diagnostics by leveraging the unique properties of nanomaterials. Gold nanoparticles, in particular, have been widely used for signal amplification and detection in various biochemical assays due to their ability to conjugate with antibodies [12–15]. In the current study, the incorporation of gold nanoparticles conjugated with the detection antibody in the nano-based ELISA resulted in enhanced signal amplification, contributing to the improved performance observed. The nano-based ELISA demonstrated high sensitivity and specificity, with no cross-reactivity observed against serum samples from animals infected with other helminth parasites, such as *G. intestinalis, P. equorum*, and *O. equi*. This high specificity is crucial for accurate diagnosis, as it ensures that the assay specifically detects antibodies against S. vulgaris without interference from other parasitic infections.

Moreover, the nano-based ELISA exhibited a positive correlation between the optical density (OD) readings and the intensity of S. vulgaris infection, as determined by the egg per gram (EPG) counts. Higher OD values were observed in animals with higher EPG counts, indicating a stronger antibody response against the *S. vulgaris* excretory-secretory (E/S) antigen in more severe infections. This quantitative relationship between OD readings and infection intensity could potentially aid in monitoring the progression or regression of the disease and assessing the efficacy of therapeutic interventions.

The selection of an appropriate cut-off value is crucial for accurate interpretation of ELISA results. In this study, the cut-off values were determined based on the mean OD of negative control sera plus three times the standard deviation, a widely accepted approach in ELISA studies [19]. The use of proper techniques for antigen extraction and purification is also essential for maintaining the high specificity and sensitivity of the ELISA, as highlighted by the increasing adoption of E/S-ELISA in seroepidemiological investigations [27].

Early detection of *S. vulgaris* infections in farmed horses is critical for implementing effective control strategies. Immunological diagnostic techniques like ELISA offer a rapid and accurate screening method for large numbers of samples, making them valuable tools in equine medicine [27]. The integration of nanotechnology, as demonstrated in this study, further enhances the sensitivity and specificity of i-ELISA, aligning with findings from other studies that have employed nanoparticle-based ELISA for human disease diagnostics [28–32]. However, these diagnostic techniques are seldom employed in the detection of diseases of domestic horses. According to Zhang et al. [33], nanoparticle-based ELISA approaches enable earlier diagnosis of disease-causing agents, detect targeted disease biomarkers at lower levels with high accuracy, and improve treatment monitoring. These advantages are particularly relevant in the context of *S. vulgaris* infection, where early detection of migratory larvae is crucial for preventing the development of verminous aneurysms and associated clinical complications.

The application of nano-biotechnology in disease diagnostics has gained significant momentum in recent years [34–37], offering promising avenues for enhancing established diagnostic approaches. However, studies exploring the potential of nanotechnology in parasitology remain limited. The findings of the present study demonstrate the feasibility and benefits of employing a nano-based ELISA for serodiagnosis of *S. vulgaris* infection in horses. In the absence of commercially available ELISA kits specific for horses, the development of a highly sensitive and specific nano-ELISA using E/S antigens can facilitate comprehensive evaluation of immune responses and epidemiological studies in horses [38–41] Furthermore, this approach holds promise for future research aimed at improving the identification of other parasitic diseases, including verminous arteritis caused by immature *S. vulgaris* worms, which can lead to life-threatening embolisms in the cranial mesenteric artery.

## Conclusions

Our study successfully developed a highly sensitive and specific nano-gold ELISA for early diagnosis of *S. vulgaris* in horses. This novel approach surpassed i-ELISA, showing improved signal amplification, higher specificity, and a quantitative link between optical density and infection intensity. These findings illuminate the potential of nanotechnology in parasitological diagnostics, paving the way for improved disease management and enhanced animal well-being. This nano-gold ELISA offers rapid, specific, and sensitive detection of *S. vulgaris*, making it a valuable tool for screening horse populations during outbreaks and preventing life-threatening complications. Additionally, its quantitative capabilities enable disease progression monitoring and treatment efficacy evaluation, facilitating optimal management strategies. Finally, this study presents a significant advancement in parasitological diagnostics with the nano-gold ELISA. Its superior performance and quantitative insights hold promise for effective control of *S. vulgaris* infections in horses and encourage further exploration of nanotechnology's potential in addressing critical challenges in veterinary medicine.

## Data Availability

All data generated or analyzed during this study are included in this article.
